# The effectiveness of a training programme in everyday cognition in healthy older adults: a randomised controlled trial

**DOI:** 10.1186/s12877-020-01998-7

**Published:** 2021-01-28

**Authors:** Celia Sánchez Gómez, Eduardo José Fernández Rodríguez

**Affiliations:** 1 Department of Evolutionary and Educational Psychologyt, Faculty of Psychology. University of Salamanca, Avenida de la Merced, 109, 37005 Salamanca, Spain; 2 Department of Nursing and Physiotherapy, University of Salamanca, Salamanca, Spain; 3 Biomedical Research Institute of Salamanca, Salamanca, Spain

## Abstract

**Background:**

Everyday cognition is the application of basic cognitive skills and knowledge of the specific cognitive domain for the resolution of problems that are integrated within the instrumental domains of functioning. The main objective is to evaluate the effectiveness of a *Training Programme in Everyday Cognition* in order to improve the levels of everyday cognition and global cognitive performance in older adults.

**Methods:**

A randomised controlled trial of two groups. The sample was composed of healthy older adults. The intervention of the experimental group consisted of an *Everyday Cognition Training Programme*, and the intervention of the control group consisted of a *Conventional Cognitive Training Programme*. The Rapid Assessment of Cognitive Functions test (ERFC) and the Everyday Cognition Battery test (ECB) were used to assess the intervention.

**Results:**

Total sample (*n* = 237) composed of 44 men and 223 women, with a mean age of 73.45 years. Statistically significant differences (*p < 0.001*) were evidenced between the control group and the experimental group in both the ECB and ERFC; in the final evaluation of the study and in the follow-up.

**Conclusion:**

The use of a *Daily Cognition Training Programme* presents greater benefits in terms of both *global cognitive performance* and *everyday cognition* than the use of a *Conventional Cognitive Training Programme* in elderly adults.

**Trial registration:**

ClinicalTrials.gov: NCT04041999.

Retrospectively registered. Date of trial registration: 8th July 2019.

## Background

In recent years, thanks to advances in medical techniques, life expectancy has increased greatly. As a result, the longevity of the population has led to a progressive increase in the incidence and prevalence of degenerative diseases such as mild cognitive impairment (MCI) and dementia [1, 2]. Given the high number of older adults worldwide, the study of cognitive functions is gaining considerable importance. In general, dementias are among the diseases of greatest clinical and health concern. However, there is a population group of older adults with undiagnosed cognitive impairment. MCI is still under-diagnosed and under-estimated, which makes these elderly people a population group at risk of dementia [3]. This is due to the fact that studies conclude that individuals with MCI have a high risk of progressing to dementia [4, 5], generally Alzheimer’s disease. Different research studies have tried to explore this issue through prospective and longitudinal studies [5, 6], in which they compare groups of older adults with and without MCI over time, observing, that people with MCI present a higher risk of developing dementia than people who do not have MCI; and furthermore the more years of follow, the greater the probability of converting MCI into dementia. Therefore, MCI is a potential risk factor for the development of dementia [7]. Based on the previous evidence, we believe in the importance of early diagnosis and intervention in healthy older adults, to achieve normal cognitive aging and prevent MCI.

To achieve active aging and a good quality of life, cognitive evaluations and interventions should be based on the impact of cognition on occupational functioning and performance, rather than on the evaluation of the intelligence and cognitive potential of individuals [8]. Everyday cognition consists of the application of basic cognitive skills and knowledge of the specific cognitive domain for the resolution of problems that are integrated within the instrumental activities of functioning [9, 10]. Currently, there is no biomarker that facilitates the diagnosis of MCI or prodromal phase of dementia and determines the prognosis, therefore early diagnosis in clinical practice relies on neuropsychological assessment [11, 12].

Generally, cognitive screening tests or brief cognitive tests [13–15] are used to evaluate a patient suspected of having cognitive impairment [16]. There are many instruments for assessing cognitive functions. However, not many do it by analysing the functional aspect [8]. It should be noted that the ability to successfully solve instrumental real-world problems is directly associated in the elderly with the ability to remain independent [17]. Without a doubt, this functional independence and personal autonomy translates into a greater self-perception by the older adult in terms of quality of life. Therefore, different researchers have argued that it is important to assess everyday cognition, instead of assessing cognitive performance with conventional measures, which are often out of context and not very objective [18]. For this purpose, there are tests that present cognitively challenging everyday problems and that have been designed to measure the ability to solve tasks related to instrumental activities of daily living (IADL) [19].

Regarding cognitive interventions in older adults, the main objective has always been to stop, control or slow down the progression of cognitive impairment through different pharmacological therapies [20]. However, at present, numerous investigations highlight the role, both preventive and therapeutic, of non-pharmacological therapies in older adults [21]. Among Non-Pharmacological Therapies, cognitive *training* has proved to be one of the most valid. Moreover, it plays a fundamental role due to its effects on the cognition and functionality of individuals [21].

Cognitive training is established as an intervention [22] that can be used both with a *therapeutic objective* in older adults with some type of cognitive deterioration [23] and with a *preventive objective*, in healthy older adults [24]. In this case, the objectives are to develop mental capacities, to improve and to optimize their functioning. Taking into account that the objective of any cognitive training programme should not only be the improvement or maintenance of the basic cognitive functions, but also the generalisation and transference of that improvement to the elderlies’ daily life, we propose a mode of action based on an *“Everyday Cognition Training Programme”*, as well as a specific assessment of everyday cognition. Specifically, we will focus on the correct intake of medication and adherence to medical treatment, giving total priority to the gain in terms of independence.

Labra Pérez et al. [25] carried out a study on the importance of the participation of older adults in cognitively demanding daily activities. The aim of the research was precisely to analyse the relationship between daily stimulation and cognitive functioning. The results showed that various cognitively demanding everyday tasks are related to cognitive processes. They also evidenced the importance of everyday activity as a protective mechanism against cognitive decline, together with the need to maintain an active ageing. In the existing literature, we find that there is significant inter-individual variability in cognitive ageing [26]. The frequency and content of the cognitive training are among the related factors [27]. Several studies have shown that older adults who participate in intellectual activities, cultural events or cognitive training programmes can slow down their cognitive decline or maintain cognitive function [28].

The main objective of our study is to evaluate the effectiveness of an “*Everyday Cognition Training Programme”* as a novel tool for cognitive *training* in the elderly, to improve levels of everyday cognition and global cognitive performance. Another secondary objective is to analyse, whether there is a difference between the aforementioned programme and a “*Conventional Cognitive Training Programme”* in terms of levels of everyday cognition and cognitive performance in older adults and to study the relationship between standard psychometric tests which measure cognitive performance and the Declarative Memory part of the Everyday Cognition Battery Recognition Test (ECB)”, which measures everyday cognition.

## Methods

### Trial design

Experimental, randomised, stratified, prospective, longitudinal study using a fixed-assignment parallel scheme with an experimental group and a control group.

### Participants

Healthy older adults of both sexes, who voluntarily completed an Occupational Therapy Programme organised by the University of Salamanca. The programme was implemented in their corresponding Day Centre or Social Association for older people, during the years 2014–2018, and the participants met the following selection criteria*.*

#### Inclusion criteria

To be aged 60 or, to perform the initial assessment of the first stage of the study (A-1) and to voluntarily authorise their participation in the study by signing the informed consent.

Exclusion criteria: To present cognitive impairment with a clinical diagnosis, be illiterate, not authorising their participation in the study, not meeting the inclusion criteria, not participating in another cognitive training programme on a regular basis and not carrying out the initial assessments of any of the four stages of the study.

Withdrawal criteria: Not performing the final assessments of any of the four stages of the study, not continuing in the study of their own free will and to quit the Occupational Therapy Programme.

ORIGIN OF THE PARTICIPANTS: Ten municipal centres and associations for older people, assigned to the City Council of Salamanca (Spain).

### Interventions

To evaluate the effectiveness of an *“Everyday Cognition Training Programme”* as a novel tool for cognitive training in the elderly and to analyze whether there is a difference between the aforementioned program and a *“Conventional Cognitive Training Programme”.*

**Fig. 1:**
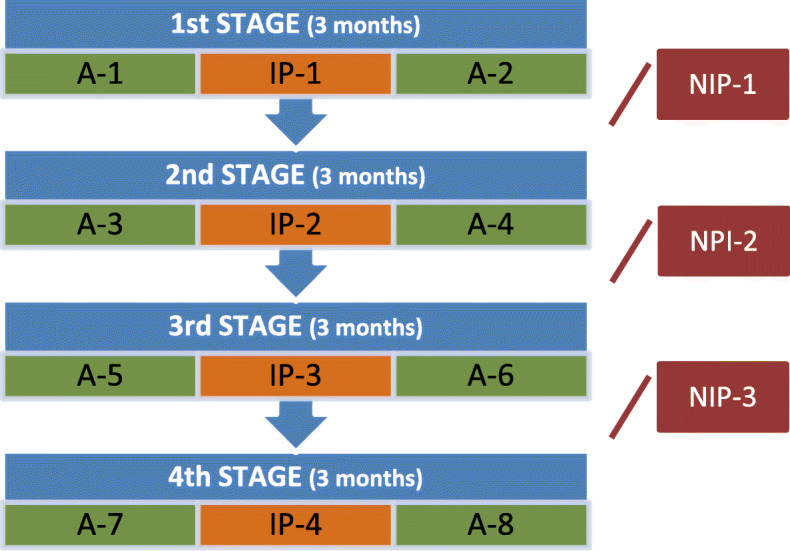
*Study sequence.* Graphic description of the process carried out throughout the development of the studywhere we can observe the sequencing of the evaluations and interventions of the 4 stages carried out during the 4 years of study duration. *(A-1) Initial Assessment in 1st Stage or Assessment 1; (IP-1) Intervention Phase 1; (A-2) Assessment 2 or Final Assessment in 1st Stage; (NIP-1) Non-Intervention Phase 1; (A-3) Assessment 3 or Initial Assessment in 2nd Stage; (IP-2) Intervention Phase 2; (A-4) Assessment 4 or Final Assessment in 2nd Stage; (NIP-2) Non-Intervention Phase 2; (A-5) Assessment 5 or Initial Assessment in 3rd Stage; (IP-3) Intervention Phase 3; (A-6) Assessment 6 or Final Assessment in 3rd Stage; (NIP-3) Non-Intervention Phase 3; (A-7) Assessment 7 or Initial Assessment in 4th Stage; IP-4: Intervention Phase 4; (A-8) Assessment 8 or Final Assessment*

The development of the study over four years (2014–2018) was as follows (Fig. 1):

1. After being admitted to the Occupational Therapy Programme, meeting the selection criteria and signing the informed consent, the groups were randomised.
2. **Initial assessment or Assessment 1 (A-1)**: before the intervention. It consisted in the documentation of the clinical history and the performance of the tests (Battery ECB and Questionnaire ERFC).
3. **Intervention Phase 1 (IP-1)**: in each intervention phase, 20 sessions were carried out in each group; two sessions per week were conducted with an approximate duration of 3 months.
4. After the intervention, **Assessment 2 or Final Assessment in 1st Stage (A-2)** was performed, with the same tests as in A-1.
  The time between A-1 and A-2 is the **1st STAGE.**
5. After A-2, a period was established in which participants did not receive intervention. We call this period the **Non-Intervention Phase 1 (NIP-1).**
  Each non-intervention phase lasted approximately one year.
6. The **2nd STAGE** began with **Assessment 3 or Initial Assessment in 2nd Stage (A-3**), followed by **Intervention Phase 2 (IP-2),** and ended with **Assessment 4 or Final Assessment in 2nd Stage (A-4).**
7. The process continued with the **Non-Intervention Phase 2 (NIP-2)**, which gave way to the **3rd STAGE**. This stage began with **Assessment 5 or Initial Assessment in 3rd Stage (A-5),** continued with the **Intervention Phase 3 (IP-3)** and ended with **Assessment 6 or Final Assessment in 3rd Stage (A-6).**
8. It continued with the **Non-Intervention Phase 3 (NIP-3**), reaching the last stage of the study, the **4th STAGE**. This stage is comprised of **Assessment 7 or Initial Assessment in 4th Stage (A-7),** an **Intervention Phase 4 (IP-4)**, and the last assessment which is **Assessment 8** or **Final Assessment or Final Assessment (A-8).**

All the interventions were carried out by an occupational therapist throughout the 4 years and therefore of the 4 stages of the study. This occupational therapist is a professor and researcher at the University of Salamanca as well and has experience in this type of intervention aimed at maintaining / improving cognitive functions.

The interventions were carried out face-to-face in municipal centres and associations for older people, assigned to the City Council of Salamanca. The rooms of the centers for the elderly where the interventions were carried out were similar in terms of infrastructure. All of them have tables and chairs for all participants, good acoustics and light and a suitable environment free of distracting stimuli.

**Fig. 2:**
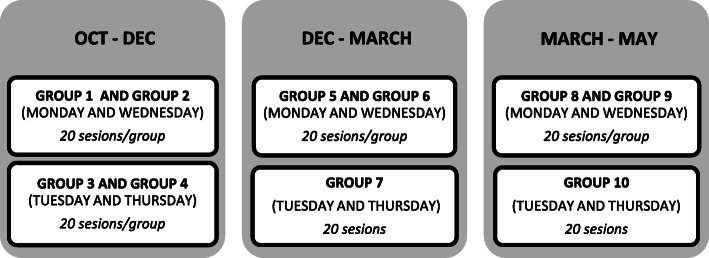
*Time distribution of the Occupational Therapy Programme groups in each stage of intervention.* Development of the temporality carried out with the groups of participants of the occupational therapy program in the 4 intervention phases. Specifying when each group performed the intervention, duration, frequency and number of sessions

The intervention was organised by the University of Salamanca in 10 groups/day centre, of which 5 belonged to the experimental group and 5 to the control group. Each group consisted of a maximum of 25–30 participants. Although the explanation of the tasks carried out in the sessions was given to the entire group, each participant had to do it individually afterwards. In each of the 10 groups, *20 intervention sessions* with a duration of 50 min were carried out (2 sessions/week on alternate days in the morning. Figure 2 shows the distribution in time of the groups. All the participants who completed the 4 stages of the study, received a total of *80 sessions.*

Although the number of sessions and their duration was the same in the two groups; the sessions differed in terms of the procedures used, a different intervention program was used in each of the study groups:

A. **Control group:** An intervention based on a “*Conventional Cognitive Training Programme*” was carried out.

- In the *“Conventional Cognitive Training Programme”*, tasks were carried out to exercise various cognitive functions: orientation, gnosis, executive functions (mainly working memory, decision making, planning, reasoning and time estimation), praxis, attention, language and memory.
  The material used in the “*Conventional Cognitive Training Programme*” was mainly stationery (sheets, pens, pencils, etc.). In addition, in some sessions computers were used, which were available in all the centers for older people.

B. **Experimental group:** An intervention based on an *“Everyday Cognition Training Programme”* was conducted.
  In the *“Everyday Cognition Training Programme”*, we focused on tasks related to **medication taking and adherence to treatment**, due to the great importance of this activity in older adults, the difficulty it sometimes implies for them and the various consequences that incorrectly taking medications could entail in such an essential IADL. All the activities performed involved the application of cognitive processes such as working memory, processing speed, attention, reasoning or planning.
  Some of the tasks carried out were: handling a medical prescription; controlling the expiration of medications; understanding medical prescriptions, guidelines for the correct taking of medication (dose, schedules...) and indications and contraindications; filling pill boxes; recalling medical check-ups; and prospective memory of medical management.
  For this purpose, materials similar or equal to those that the older adult could find in daily tasks or when facing the resolution of daily problems were used, thus bringing the intervention closer to real life. For example, medication pill boxes, documents designed to be as close to reality as prescription drugs, package leaflet of the medicinal product, medical reports, follow-ups and medical appointments, etc.

The big difference between an *Everyday Cognition Training Programme* (experimental group) and a *Conventional Cognitive Training Programme* (control group), is that in the former the participants exercise different cognitive functions during the development of different IADLs; using *‘Everyday functioning’* as the task domain. Whereas in the latter participants using *‘Cognitive functioning’* as the task domain, exercising these cognitive functions by performing tasks that are far from being able to be generalised to the daily routine.

The intervention was not modified during the course of the study, which was based on the previous performance of a pilot study.

### Outcome measures

#### Description of the variables under study

- DEPENDENT VARIABLES:
- Everyday Cognition: measured by the Everyday Cognition Battery (ECB).
- Cognitive performance: measured by the Rapid Assessment of Cognitive Functions (ERFC).
- INTERVENING VARIABLES: Age, gender and level of education.

#### Measures

Each participant performed a total of 8 assessments, 2 (initial and final) for each of the 4 stages of intervention. The first assessment of the study was conducted at the beginning of the Occupational Therapy Programme for the period 2014–2015, and the last assessment corresponds to the end assessment of the programme for the period 2017–2018.

*DEPENDENT VARIABLES:* For the dependent variables, in both groups, the participants were evaluated with two hetero-administered questionnaires:

- **Everyday Cognition Battery (ECB)** [9, 17, 18]**:**
  It should be noted that little use has been made in Spain of this type of measure. Among the existing assessment measures, we have selected the ECB (Everyday Cognition Battery) for our study.
  The ECB is a test intended for the evaluation of everyday cognition in the elderly without cognitive impairment. This questionnaire assesses cognitive competence in three instrumental domains of daily life: medication use, financial management and planning, and nutrition and meal preparation. These instrumental tasks have been described as universal, basic, and mandatory, since it is assumed that most older adults have acquired knowledge and substantial experience in these domains. Daily cognitive tasks are drawn from the broader set of IADL (Lawton & Brody, 1969) [29], a set of tasks in which older adults frequently participate in their daily lives. These are tasks that older adults are expected to perform well in order to maintain independent functioning in the real world [30]**.**
  The ECB Battery includes the following traditional psychometric measures: inductive reasoning, knowledge, declarative memory, and working memory.
  Within the ECB, there are 4 tests, each designed to assess a single cognitive ability: ECB Inductive Reasoning Test, ECB Knowledge Test, ECB Computation Span Test (Working Memory) and ECB Recognition Test (Declarative Memory).

For the study, we selected the last, since it evaluates memory, which led us to think about the importance of memory in older adults, both measured objectively and subjectively. Within the ECB Recognition Test, we focused on tests that assessed taking medication and adherence to treatment.

The ECB Battery scales between 0 and 10, that is, it has a maximum score of 10. The lower the score, the lower the cognitive performance during the development of daily activities.

**Rapid Assessment of Cognitive Functions (ERFC)** [31]**:**

This test evaluates cognitive ability and allows a quick assessment and early diagnosis of a possible cognitive deficit. It consists of 13 subtests that measure the following cognitive functions: temporospatial orientation, attention and memory (explores the *attention span*, *immediate memory* and *working memory* and, furthermore, *memory*, which examines long-term learning capacity, without help or through induced memory, consisting of offering semantic clues to words not freely remembered.), mental calculation (explored through two subtractions), reasoning and judgment, similarities (evaluates the capacity for abstraction), comprehension (specifically assesses listening comprehension), naming (explored through the naming of two real objects and two images), repetition, written order (evaluate written comprehension), verbal fluency (examines *semantic fluency* and, furthermore, *alternate phonetic fluency*), praxis (studies the symbolic gesture or *ideomotor praxia* and *constructive praxia*), visual recognition (assesses visual gnosia) and writing (explored through the copying and dictation of two words).

The ERFC Questionnaire has a maximum score of 56, except for illiterate participants, whose maximum score is 51, once the subtests of mental calculation, written order and writing have been eliminated, which require that the evaluated participants have numerical and literacy skills.

The cut-off point for the ERFC that indicates a possible cognitive impairment is located at 51 out of 56 (with a sensitivity of 0.92 and a specificity of 0.86) and at 46 points out of 51 for the group of illiterate subjects (with a sensitivity of 0.9 and specificity of 0.88).

As in the present study one of the exclusion criteria would be not having numerical and literacy skills, the group of illiterate participants would not be included, so the maximum score in our case is 56 points.

*INTERVENING VARIABLES:* A register sheet containing personal details, along with the level of studies and the day centre to which they were assigned, was designed*.*

### Sampling size

Participants of the study were recruited by convenience sample. The study sample was made up of all the users from the different day centres or associations for older people enrolled in the Occupational Therapy Programme who met the selection criteria and who authorised their participation in the study on a voluntary basis.

The sample size estimation was also based on the sample size reported by the author for the validation of the ECB Battery (with an initial sample of 174 participants and a final sample of 114 participants) [9]**.**

### Randomisation

For the randomization procedure, since each participant had to enrol in his or her corresponding day centre for older people and all participants who enrolled received intervention under the Occupational Therapy Programme, it was not possible to randomise participants, so group randomisation was performed instead.

The process of randomising the groups of participants was conducted with respect to the order of the centres where the programme was carried out, by the method of simple random assignment. This was done using a table of random numbers, which was generated by a researcher external to the study. Using this table, the centres that obtained an even number were assigned to the experimental group and the centres that obtained an odd number were assigned to the control group.

### Masking

The assessments were conducted by five qualified occupational therapists, of whom only one subsequently performed the intervention. In this way, 80% of the evaluations were carried out by an external evaluator, to control, as far as possible, interference or bias in the results.

Furthermore, except for the professional who performed the interventions, the rest of the evaluators did not know whether the participants belonged to the control group or to the experimental group. As for the participants, they remained blinded for the entire duration of the study.

### Statistical analysis

The variables of the study were analysed by the statistics of *Shapiro-Wilk* and *Kolmogorov-Smirnov* to know the normality of the sample at the beginning of the study, therefore determining the path to follow. The verification of the assumption of normality, according to both tests, oriented the calculations by a non-parametric route (*p* < 0.05).

Given the result of the study of normality of the sample (non-parametric analysis), for the descriptive analysis of the socio-demographic characteristics and the scores of each of the tests used, the variables were described with the corresponding statistics, using the median as a measure of centralisation and the interquartile range.

To understand the psychometric properties of the scales, Cronbach’s alpha coefficient, a factor analysis and a Pearson correlation were used.

Given the complexity of the study, follow-up comparisons of participants over time (*repeated measurements*) and/or of different groups (*independent groups*) were scheduled.

Firstly, and before carrying out the comparison of ranks through change of score, we studied whether all the initial conditions were similar between the groups to study. For this, the *Mann-Whitney U Test* or the *Kruskal-Wallis Test* were used, with equality when p>0,05.

*Comparisons of two ranks* were resolved with the *Mann-Whitney U Test* (independent groups) or with the *Wilcoxon T-test* (repeated measurements).

*Comparisons of three or more averages* were analysed with the *Kruskal-Wallis H Test* (independent groups) or with *Friedman’s Q Test* (repeated measurements).

The *correlation analysis* was solved with the *Spearman’s rank correlation coefficient* (Spearman’s rho).

This study is presented following the guidelines of CONSORT.

## Results

**Fig. 3:**
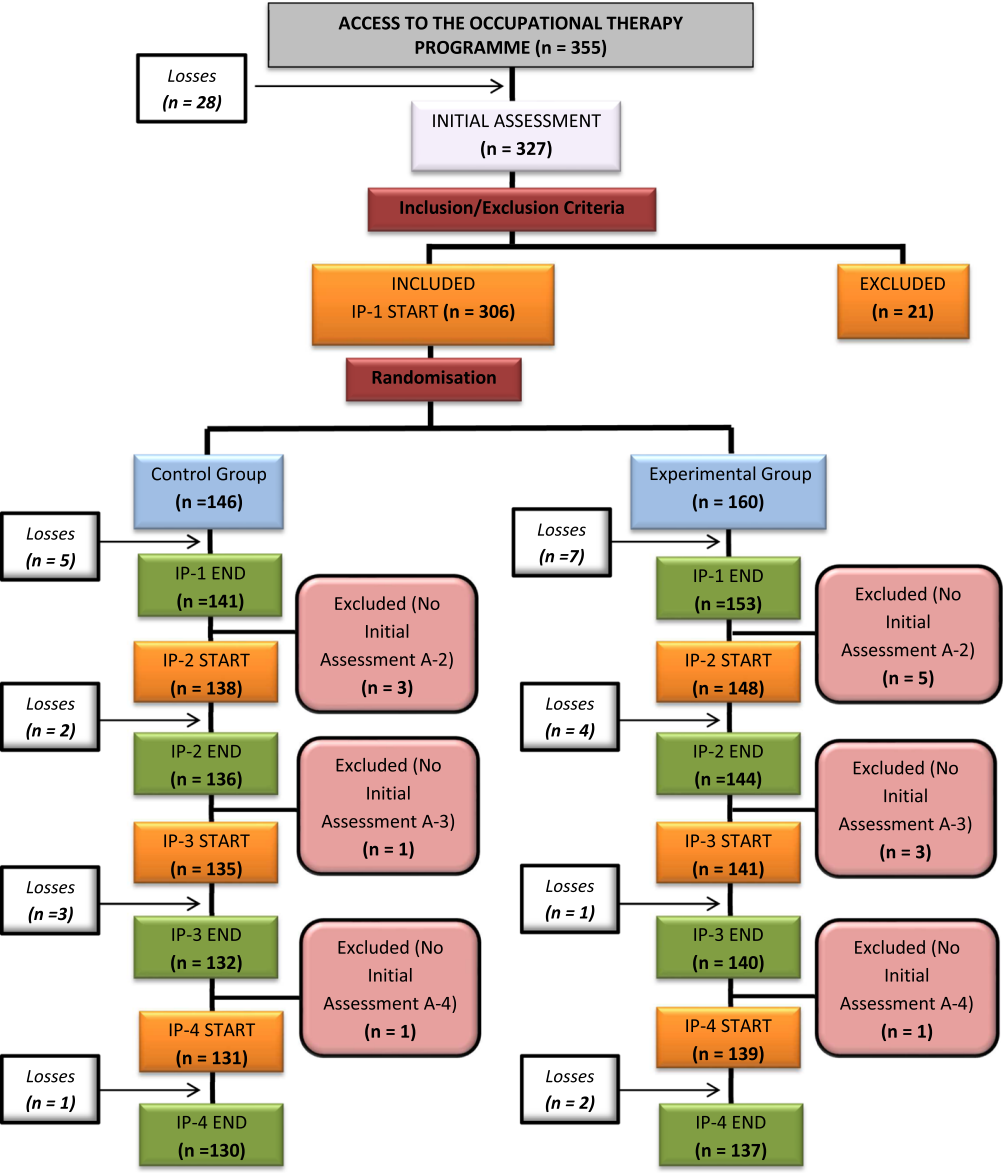
*Flow diagram of the sample of participants throughout the development of the study.* The participant flow shows the evolution of the participants from the initial sample. The number of participants who were randomly assigned, received the intended treatment, and were analyzed for the primary outcome. In addition to the drop out and exclusions after randomization for different reasons stated in the exclusion and withdrawal criteria

The study had a final sample of 237 individuals: 137 individuals in the experimental group and 130 individuals in the control group (Fig. 3).

The sample (*n* = 237) is composed of 44 men and 223 women, and the mean age of the total sample is 73.45 years (± 6.45). Regarding their level of education, most of the participants have at least primary education (78.70%) (refer to the table Table 1).

**Table 1:** Descriptive statistics – Socio-demographic data

		EXPERIMENTAL GROUP (***n*** = 137)		CONTROL GROUP (***n*** = 130)		TOTAL SAMPLE (***n*** = 237)	
**AGE**		73.89		72.99		73.45	
**GENDER**	MALE	22	16.10%	22	16.90%	44	16.50%
	FEMALE	115	83.90%	108	83.10%	223	83.50%
**LEVEL OF EDUCATION**	PRIMARY	107	78.10%	103	79.20%	210	78.70%
	SECONDARY	21	15.30%	8	6.20%	29	10.90%
	HIGHER ED.	9	6.60%	19	14.60%	28	10.50%

The table shows the descriptive statistics of the sociodemographic variables of the total sample and of both study groups

We observe there are no between-group differences on any measure at baseline *(*Table 1*):* The experimental group (*n* = 137) is composed of 22 men and 115 women, with a mean age of 73.89 years (± 6.38). In the experimental group 78.10% of the participants had primary studies, 15.30% secondary studies and 6.60% higher studies. The control group (*n* = 130) is composed of 22 men and 108 women, with a mean age of 72.99 years (± 6.51). In the control group 79.20% of the participants had primary studies, 6.20% secondary studies and 14.20% higher studies.

Table 2 shows the results of the descriptive statistics of the scores obtained in the *ERFC Questionnaire* and the *ECB Battery* in the 8 evaluations carried out. By analysing the ERFC scores, it can be observed that in the 4 stages, in both groups, there was an increase in the scores after the intervention, but in the experimental group the increase was greater. The same applies to the ECB Battery, but in this case, the increase in the score was notably greater in the experimental group than in the control group.

**Table 2:** Descriptive statistics of the ERFC Questionnaire and the ECB Battery of the Experimental group and the Control group

	STAGE	ASSESSMENT	EXPERIMENTAL GROUP			CONTROL GROUP		
			m ± SD	M	IQR	m ± SD	M	IQR
**ERFC**	**1st STAGE** **2014–2015**	ERFC 1-PRE	49.63 ± 3.21	50.00	5.0	50.02 ± 3.25	50.00	4.5
		ERFC 2-POST	53.16 ± 1.91	53.50	2.8	51.85 ± 2.71	52.00	4.0
	**2nd STAGE** **2015–2016**	ERFC 3-PRE	49.70 ± 2.83	50.00	4.0	50.19 ± 3.37	51.00	4.5
		ERFC 4-POST	53.36 ± 1.78	54.00	3.0	52.08 ± 2.95	53.00	3.5
	**3rd STAGE** **2016–2017**	ERFC 5-PRE	49.96 ± 2.80	50.00	4.0	50.36 ± 3.31	51.00	4.5
		ERFC 6-POST	51.71 ± 1.74	52.00	2.5	51.99 ± 2.88	52.50	4.0
	**4th STAGE** **2017–2018**	ERFC 7-PRE	50.21 ± 2.88	50.00	3.8	50.45 ± 3.26	51.00	4.0
		ERFC 8-POST	53.97 ± 1.84	54.50	2.8	52.28 ± 2.77	52.75	3.3
**ECB**	**1st STAGE** **2014–2015**	ECB 1-PRE	5.08 ± 2.07	4.00	2	5.24 ± 1.90	6.00	2
		ECB 2-POST	8.46 ± 1.46	8.00	2	6.00 ± 1.97	6.00	4
	**2nd STAGE** **2015–2016**	ECB 3-PRE	5.19 ± 2.00	6.00	2	5.35 ± 1.86	6.00	2
		ECB 4-POST	8.49 ± 1.38	8.00	2	6.12 ± 1.90	6.00	4
	**3rd STAGE** **2016–2017**	ECB 5-PRE	5.14 ± 2.11	6.00	2	5.20 ± 2.19	6.00	2
		ECB 6-POST	8.49 ± 1.45	8.00	2	6.03 ± 2.02	6.00	4
	**4th STAGE** **2017–2018**	ECB 7-PRE	5.48 ± 2.12	6.00	4	5.21 ± 2.32	6.00	2
		ECB 8-POST	9.02 ± 1.39	10.00	2	5.84 ± 2.16	6.00	4

The descriptive statistics results table shows the scores obtained in the ERFC Questionnaire and the ECB Battery, in the 8 evaluations carried out throughout the 4 study stages, in the experimental group and in the control group. We can observe the scores before and after the intervention in both questionnaires

To check whether the implementation of different interventions in each of the groups produced differences in the cognitive performance and everyday cognition of the participants, the following actions were taken:

- First, a **comparison in both groups separately** of the mean scores of the *initial and final assessments* was made. These mean scores took into account both the initial assessment made at the beginning of the study **(1-PRE)** and the last assessment made at the end of the study **(8-POST)**. **The initial and final assessments at each stage of the study** were also taken into account, both from the ERFC Questionnaire and the ECB Battery (Table 3). Statistically significant differences were found in both the control group and the experimental group between the 1-PRE and 8-POST assessment, as well as between the initial and final assessments at each stage of the study in “ERFC” (*p* < 0.001) and “ECB” (*p* < 0.001).
  Observing these results, we can point out that in both groups there was an increase in 8-POST with respect to 1-PRE, as well as in all stages of intervention. Although the Z value in the experimental group is notably higher, especially in the ECB, we do not know the amount of this increase, nor can we say whether one group increases more than the other.
- Secondly, the **comparison between the two groups** was made. For this purpose, the variables “difference between **1-PRE and 8-POST**” and “difference between the **initial and final assessment of each of the 4 stages of the study**” were generated. As analysed in Table 3, in the case of ERFC, ***statistically significant differences were obtained between the control group and the experimental group*** in the *variable difference 1-PRE and 8-POST* (*p* < 0.001) and in the variable *difference between the initial and final assessments* in all the stages of the study (*p* < 0.001), except in the 3rd stage (*p* = 0.431). In the case of ECB, statistically significant ***differences were obtained between the control group and the experimental group, in all variables under study*** (*p* < 0.001).

**Table 3:** Comparison of ranks between initial and final evaluations in the CONTROL GROUP and in the EXPERIMENTAL GROUP; and comparison between the CONTROL GROUP-EXPERIMENTAL GROUP

**Comparison of ranks**					
	**VARIABLES PER STUDY STAGE**	***CONTROL***		***EXPERIMENTAL***	
		**Z**	**p**	**Z**	**p**
**ERFC**	**ERFC (1-PRE) – ERFC (8-POST)**	−8.12	***.000***	−10.00	***.000***
	ERFC (2-POST) – ERFC (1-PRE)	−9.75	***.000***	−10.13	***.000***
	ERFC (4-POST) – ERFC (3-PRE)	−9.79	***.000***	− 10.17	***.000***
	ERFC (6-POST) – ERFC (5-PRE)	−9.70	***.000***	−8.69	***.000***
	ERFC (8-POST) – ERFC (7-PRE)	−9.73	***.000***	−10.06	***.000***
**ECB**	**ECB (1-PRE) – ECB (8-POST)**	−3.78	***.000***	−9.73	***.000***
	ECB (2-POST) – ECB (1-PRE)	−6.09	***.000***	−10.20	***.000***
	ECB (4-POST) – ECB (3-PRE)	−6.16	***.000***	−10.25	***.000***
	ECB (6-POST) – ECB (5-PRE)	−6.71	***.000***	−10.24	***.000***
	ECB (8-POST) – ECB (7-PRE)	−6.04	***.000***	−10.30	***.000***
**Comparison** ***CONTROL- EXPERIMENTAL***					
	**VARIABLES PER STUDY STAGE**	**U**		**p**	
**ERFC**	**ERFC DIFFERENCE (1PRE-8POST)**	5080.00		***.000***	
	ERFC DIFFERENCE (1PRE-2POST)	3865.50		***.000***	
	ERFC DIFFERENCE (3PRE-4POST)	3275.00		***.000***	
	ERFC DIFFERENCE (5PRE-6POST)	8413.00		***.431***	
	ERFC DIFFERENCE (7PRE-8POST)	2995.00		***.000***	
**ECB**	**ECB DIFFERENCE (1PRE-8POST)**	2509.00		***.000***	
	ECB DIFFERENCE (1PRE-2POST)	1940.00		***.000***	
	ECB DIFFERENCE (3PRE-4POST)	1959.00		***.000***	
	ECB DIFFERENCE (5PRE-6POST)	1954.00		***.000***	
	ECB DIFFERENCE (7PRE-8POST)	1287.00		***.000***	

The table shows the comparison of ranks between initial and final assessments in the 4 stages of the study; and in the 8-POST and 1-PRE assessment in ERFC Questionnaire and ECB Battery in the CONTROL GROUP and in the EXPERIMENTAL GROUP; and comparison between CONTROL GROUP-EXPERIMENTAL GROUP of the variable “difference of the 4 stages of the study” and the variable “difference 1-PRE - 8-POST” of the ERFC Questionnaire and ECB Battery

*(1-PRE) Initial Assessment in 1st Stage; (2-POST) Final Assessment in 1st Stage; (3-PRE) Initial Assessment in 2nd Stage; (4-POST) Final Assessment in 2nd Stage; (5-PRE) Initial Assessment in 3rd Stage; (6-POST) Final Assessment in 3rd Stage; (7-PRE) Initial Assessment in 4th Stage; (8-POST) Final Assessment in 4th Stage*

With regard to the *study of correlations*, we took as valid those with a positive statistical significance, with significance indices of *p* < 0.05. The results revealed **significant positive relationships between all the variables studied**, regarding the scores corresponding to ERFC and the scores corresponding to ECB *(p < 0.001*) (Table 4). Furthermore, we can add that this significance was obtained in all the situations studied, both in the initial assessment at the beginning of the study (1-PRE) and in the final assessment after 4 periods of intervention (8-PRE) (refer to the table Table 4).

**Table 4:** Analysis of the correlations between ERFC and ECB variables and Analysis of the correlations between socio-demographic variables and ERFC and ECB

	ERFC (1-PRE)	ERFC (8-POST)	ECB (1-PRE)	ECB (8-POST)
**ERFC (1-PRE)**				
ERFC (8-POST)	.566 (a)			
	***.000*** (b)			
	267 (c)			
ECB (1-PRE)	.550 (a)	.333 (a)		
	***.000*** (b)	***.000*** (b)		
	267 (c)	267 (c)		
ECB (8-POST)	.197 (a)	.641 (a)	.253 (a)	
	***.001*** (b)	***.000*** (b)	***.000*** (b)	
	267 (c)	267 (c)	267 (c)	
AGE	−.270 (a)	−.228 (a)	−.211 (a)	−.174 (a)
	***.000*** (b)	***.000*** (b)	***.001*** (b)	***.004*** (b)
	267 (c)	267 (c)	267 (c)	267 (c)
LEVEL OF EDUCATION	.325 (a)	.255 (a)	.302 (a)	.183 (a)
	***.000*** (b)	***.000*** (b)	***.000*** (b)	***.003*** (b)
	267 (c)	267 (c)	267 (c)	267 (c)

*(a) Spearman’s rho; (b) p; (c) n*

The table shows, on the one hand, the correlation of the variable “ERFC” with variable “ECB”, both with the score obtained in the Initial Assessment (1-PRE) and in that obtained in the Last Assessment (8-POST), and on the other hand, the correlation between the sociodemographic variables “Age” and “Educational Level” with the score obtained in the ERFC Questionnaire and in the ECB Battery, both with the score obtained in the Initial Assessment (1-PRE) and in the obtained in the Last Assessment (8-POST)

Furthermore, results showed that any score of the variables “ECB” and “ERFC” taken at time 1-PRE was significantly and positively correlated to any value of the same variable at time 8-POST (*p < 0.001*) (Table 4).

As for the *correlation of these variables with the socio-demographic variables*, **significance was again obtained in all the cases studied** (Table 4). On the one hand, a significant and positive correlation was observed between “Level of education” and “ERFC” “ECB”. On the other hand, the relationship with the variable “Age” was significant and negative in all the cases analysed.

## Discussion

The realization of this research arose from the need to study the evolution at a cognitive level of a population of older adults who participated in an Occupational Therapy Programme, lived in the town of Salamanca (Spain) and received cognitive *training*.

The data from our study reflect a good balance between the characteristics of both groups, having studied the initial equality of all variables in all cases. This means there were not significant differences prior to the intervention that could affect the results.

With regard to cognitive performance (*ERFC Test*) we observe that, taking into account the results achieved when analysing each group separately and comparing the initial and final scores, we can point out that in both groups there was a significant increase in the 8-POST score as compared to the 1-PRE score, as well as in the final scores as compared to the initial scores at all stages of intervention.

As can be seen, when analysing each group independently, both groups obtained an improvement in their *global cognitive performance*; this may be due to the fact that in both groups the intervention was directly aimed at the cognitive function of individuals. However, it should be noted that the experimental group obtained a greater increase. Furthermore, this data indicates that cognitive *training* in general, independently of the procedure carried out in each group, is indeed a useful tool to improve the global cognitive performance of older adults, as all the studies we have previously analysed point out [32, 33].

However, when comparing the control group against the experimental group, statistically significant differences were found in the difference between A-1 and A-8 in ERFC. These results lead us to affirm that, although it is true that the implementation of a traditional cognitive *training* programme improves the global cognitive performance in older adults, the implementation of a programme of everyday cognition seems to report greater benefits.

Regarding the analysis of everyday cognition (through ECB), results obtained were positive: it was observed that both groups improved their everyday cognition after the intervention, but clearly the individuals in the experimental group obtained a notably greater increase than those in the control group. Moreover, when comparing both groups, statistically significant differences were obtained in the difference between the *Initial Assessment* made at the beginning of the study and *Assessment 8* made at the end of the study, and also after the intervention in all the stages of the study.

These results confirm that those people who have benefited from *specific training in everyday cognition* notably improve their cognitive capacity to solve everyday problems: this is the most relevant finding we have obtained. In addition, as we have already pointed out, they also significantly improve their global cognitive performance.

As for the individuals in the control group, we can observe that there is also an improvement in their cognitive performance, consistent with the *Conventional Cognitive Training Programme* from which they have benefited, but the difference in everyday cognition is less. This leads us to wonder whether this improvement could eventually be transferred to their everyday life, since this group of individuals who did not benefit from specific training in everyday cognition might experience a functional improvement.

Another aspect to highlight is that we observed a significant and positive correlation between the ERFC scores and the ECB scores in both A-1 and A-8. Indeed, the better the cognitive performance of the individuals in our sample, the better their everyday cognition and vice versa. In addition, a positive and significant correlation was also found within each of the scales at time 1-PRE and time 8-POST. That is, between ERFC TOTAL 1-PRE and ERFC TOTAL 8-POST, and between ECB 1-PRE and ECB 8-POST.

Similar results have been described in other research, such as a prospective epidemiological study by Allaire and Willis [24], which demonstrated a relationship between both types of measures. Or in the study by Menor J et al. [25], in which, in addition to using measures of everyday cognition and global cognitive performance, they employed scales to assess specific cognitive functions (comprehension, reasoning, semantic memory, executive functions and working memory).

In addition to the indicated findings, our results inform us of the existence of a significant negative correlation between age, and cognitive performance and everyday cognition. Other works analysed show similar results [34, 35].

The opposite occurs with the level of education of individuals. With the results obtained, we can affirm that there is a positive and significant relationship between the level of studies of our older adults and their cognitive performance and everyday cognition. Menor J et al. [35], in their study on the development of an instrument to evaluate everyday cognition again found similar results to ours.

In the review of the literature, we found consensus on the importance of evaluating both functional independence [36] and cognitive status in older people [23]. However, the evidence on the use of tests that assess everyday cognition is very limited. Therefore, we believe it is important to promote the use of this type of tool. Some authors [37], make direct reference to the usefulness that this type of tests may have; specifically, in the field of geriatrics, since they can complete the geriatric exploration or establish the degree of functionality of some IADLs.

We agree that maintaining cognitive functions in the elderly is highly important [23]. However, it is indisputable that people who are cognitively capable of memorising and correctly applying the dose of medication they have to take each day, or are able to interpret without the help of another person the recommendations or contraindications of a patient information leaflet, will achieve greater personal autonomy. And we believe the same would occur with any other instrumental activities of daily living.

As noted above, studies have been conducted which have used, along with conventional measures measures of everyday cognition, such as the studies by Allaire JC and Willis SL 2006 [38] and Kennedy SW et al. 2012 [39]. There are also several studies [36, 40–42], although most outside Spain, that have studied and used different tests of everyday cognition, relating these tests to different variables. However, it should be noted that little use has been made of programmes in which a *direct intervention on everyday cognition* is carried out after cognitive assessment.

To conclude, our results lead us to think that people who have benefited from a *Conventional Cognitive Training Programme* (control group) do improve their cognitive status, but not so much their everyday cognition. On the other hand, those people who have benefited from a training programme in everyday cognition (experimental group) obtain a remarkable improvement in their global cognitive function and also in their everyday cognition [43]. Therefore, the application of an intervention focused on everyday cognition should provide more benefits in older adults when it comes to applying the gains achieved to the performance of their daily tasks or to the resolution of problems that may arise in their daily lives.

These results would support those of authors such as Allaire JC and Marsiske M [18], who have long used measures of assessment of the older adults during the resolution of complex tasks of daily life, rather than evaluations out of context and which they consider to be unobjective.

In short, we believe that assessment and intervention methods in older adults with cognitive problems or at risk should be rethought. A less theoretical and more applied approach to reality can be beneficial for them not only in terms of improving standardized cognitive outcomes, but also as a reflection in their daily lives.

We can point out as a possible limitation of the study that the assessments were carried out by five qualified occupational therapists, of whom only one carried out the intervention in both research groups. Therefore, the study cannot be referred to as double-blind, but as has been pointed out before, we tried to control for possible interference in the results by having an external evaluator perform 80% of the assessments carried out. In addition, we have observed that after the intervention phase, the participants obtained an improvement at the cognitive level. However, when they are assessed again in the next intervention phase, the gains have not been maintained. On the other hand, we have verified that they never return to the initial state before performing any treatment. It is interesting to reflect on these findings and take them into account for future research, since perhaps these types of therapies are more effective if they are used consistently and sustained over time.

## Conclusions

**Main conclusion:** The use of a *Programme of Training in Everyday Cognition* presents greater benefits in terms of both *global cognitive performance* and *everyday cognition* in older adults than the use of a *Programme of Conventional Cognitive Training*.

**Secondary conclusions:**

1. There is a significant correlation between the standard psychometric tests that measure cognitive performance and the Everyday Cognition Battery (ECB) Recognition Test (declarative memory).
2. There is a significant negative correlation between age, and global cognitive performance and everyday cognition of older adults.
3. There is a significant positive correlation between the level of education of older adults and their cognitive performance and everyday cognition.

## Data Availability

The datasets generated during and/or analysed during the current study are not publicly available due ethical restrictions but are available from the corresponding author on reasonable request.

## Abbreviations

MCI: Mild cognitive impairment
IADL: Instrumental activities of daily living
ECB: Everyday Cognition Battery
A-1: Initial assessment or Assessment 1
IP-1: Intervention Phase 1
A-2: Assessment 2
NIP-1: Non-Intervention Phase 1
A-3: Assessment 3
IP-2: Intervention Phase 2
A-4: Assessment 4
NIP-2: Non-Intervention Phase 2
A-5: Assessment 5
IP-3: Intervention Phase 3
A-6: Assessment 6
NIP-3: Non-Intervention Phase 3
A-7: Assessment 7
IP-4: Intervention Phase 4
A-8: Assessment 8 or Final Assessment
ERFC: Rapid Assessment of Cognitive Functions
1-PRE: Initial Assessment in 1st Stage
2-POST: Final Assessment in 1st Stage
3-PRE: Initial Assessment in 2nd Stage
4-POST: Final Assessment in 2nd Stage
5-PRE: Initial Assessment in 3rd Stage
6-POST: Final Assessment in 3rd Stage
7-PRE: Initial Assessment in 4th Stage
8-POST: Final Assessment in 4th Stage
IQR: Interquartile Range
m: Mean
SD: Standard Deviation
M: Median
